# Molecular Characteristics and Zoonotic Potential of *Salmonella* Weltevreden From Cultured Shrimp and Tilapia in Vietnam and China

**DOI:** 10.3389/fmicb.2020.01985

**Published:** 2020-08-25

**Authors:** Yaovi M. G. Hounmanou, Anders Dalsgaard, Tirzania Frannetta Sopacua, Gazi Md. Noor Uddin, Pimlapas Leekitcharoenphon, Rene S. Hendriksen, John E. Olsen, Marianne Halberg Larsen

**Affiliations:** ^1^Department of Veterinary and Animal Sciences, Faculty of Health and Medical Sciences, University of Copenhagen, Copenhagen, Denmark; ^2^School of Chemical and Biological Engineering, Nanyang Technological University, Singapore, Singapore; ^3^Research Group for Genomic Epidemiology, National Food Institute, Technical University of Denmark, Kongens Lyngby, Denmark

**Keywords:** *Salmonella* Weltevreden, genome comparison, non-typhoidal *Salmonella*, microbial ecology, whole genome sequencing

## Abstract

*Salmonella* Weltevreden is increasingly reported from aquatic environments, seafood, and patients in several Southeast Asian countries. Using genome-wide analysis, we characterized *S*. Weltevreden isolated from cultured shrimp and tilapia from Vietnam and China to study their genetic characteristics and relatedness to clinical isolates of *S*. Weltevreden ST-365. The phylogenetic analysis revealed up to 312 single-nucleotide polymorphism (SNP) difference between tilapia isolates, whereas isolates from shrimp were genetically more closely related. Epidemiologically unrelated isolates from Vietnam were closely related to isolates from China, e.g., 20 SNPs differences between strains 28V and 75C. In comparison with strains from other parts of the world, our environmental isolates predominantly clustered within the continental South Asia lineage, constituted mostly of strains from human stool with as low as seven SNPs difference, e.g., 30V versus Cont_ERR495254. All sequenced isolates were MLST type ST-365 and contained the major virulence-related genes encoded by the *Salmonella* Pathogenicity Islands 1–5. Ten of the isolates harbored the *Inc*FII(S) plasmid similar to the virulence genes-mediated plasmid pSPCV of *S.* Paratyphi C, and one isolate had the *Inc*Q1 plasmid on the same contig with *str*A/B, *sul*2, and *tet*A resistance genes similar to the *Inc*Q1 type, pNUC of *S.* Typhimurium. A pangenomic analysis yielded 7891 genes including a core genome of 4892 genes, with a closely related accessory genome content between clinical and environmental isolates (Benjamini *p* > 0.05). In a search for differences that could explain the higher prevalence of *S*. Weltevreden in aquatic samples, genomes were compared with those of other *Salmonella enterica* serovars. *S.* Weltevreden revealed specific regions harboring *glp*X (Fructose-1;6-bisphosphatase; class II), *rfb*C (dTDP-4-dehydrorhamnose 3;5-epimerase), and *cmt*B (PTS Mannitol-specific cryptic phosphotransferase enzyme IIA component) involved in carbohydrate biosynthesis pathways. Our study builds grounds for future experiments to determine genes or pathways that are essential when *S.* Weltevreden are in aquatic environments and microbial interactions providing survival advantages to *S.* Weltevreden in such environments.

## Introduction

*Salmonella enterica* subsp. *enterica* serovar Weltevreden is a common cause of human salmonellosis in Asian countries like China, Singapore, Thailand, and Vietnam (Aarestrup, 2003; Makendi et al., 2016; Manipura, 2016; Ferrari et al., 2019; Lin et al., 2019). *S.* Weltevreden is frequently found in seafood and aquatic environments in Asia (Bangtrakulnonth et al., 2004; Ponce et al., 2008; Noor Uddin et al., 2015; Li et al., 2017; Ferrari et al., 2019). It is also found in other food sources including broilers in China (Ren et al., 2016), poultry and pigs in Vietnam (Lettini et al., 2016; Trung et al., 2017), as well as in vegetables in Malaysia (Awang Salleh et al., 2003). The source and transmission routes of *S.* Weltevreden, when found in meat products, are unknown, but fish meal used as a protein source in livestock feed may be an important source, as *S.* Weltevreden is often found in fish and seafood (Kumar et al., 2015; Makendi et al., 2016). This underlines that *S*. Weltevreden, like *Vibrio cholerae* (Hounmanou et al., 2019a, b), might have a preference to the aquatic environment, from where it spreads to humans, domestic animals, and food products. We previously reported a close genetic relatedness of epidemiological unrelated *S*. Weltevreden isolated from cultured shrimp in Vietnam and cultured tilapia in China using pulsed field gel electrophoresis genotyping (Noor Uddin et al., 2015; Li et al., 2017) suggesting occurrence and spread of clonal strains of *S.* Weltevreden in aquatic environments.

The increasing association with *S*. Weltevreden and human salmonellosis in Southeast Asia (Aarestrup, 2003; Makendi et al., 2016; Manipura, 2016; Lin et al., 2019) correlates with its high occurrence in seafood in the region (Ferrari et al., 2019). However, little is currently known about genetic differences between *S*. Weltevreden and other serovars that favor its frequent occurrence in the aquatic environment. Comparison of *S*. Weltevreden 2007-60-3289-1, a plant isolate, with the genome of the seafood isolate SL484 showed that the genomes of both strains are very similar and no genetic marker was found to justify the aquatic life of the seafood isolate (Brankatschk et al., 2012). An increased expression of the enterotoxin *stn* gene in *S.* Weltevreden was reported when they were grown in seafood (Kumar et al., 2015); however, this expression was also observed in *S.* Typhi. Therefore, the genetic determinants of *S.* Weltevreden that favors its frequent occurrence in the aquatic environment compared with other non-typhoidal *Salmonella* remain to be described.

The present study aimed to determine the genetic characteristics and diversity of *S*. Weltevreden originating from shrimp and tilapia in Vietnam and China. We also performed a pangenomic analysis to understand the genetic relationship between environmental and clinical *S*. Weltevreden and to identify potential genetic traits associated with an aquatic occurrence of the serovar. Finally, a genome-wide comparison was performed between our *S*. Weltevreden strains and selected broad host range and host-adapted *Salmonella enterica* serovars.

## Materials and Methods

### Strain Collection

Six strains of *S.* Weltevreden (95V, 74V, 30V, 28V, 13V, 3V) recovered in 2013 from cultured shrimp in Vietnam (Noor Uddin et al., 2015) and six strains (85C, 75C, 62C, 30C, 28C, 24C) isolated in 2013 in cultured tilapia obtained from China (Li et al., 2017) were characterized by whole genome sequencing.

### DNA Extraction and Whole Genome Sequencing

DNA was extracted using the DNeasy Blood and tissue kit following the manufacturer’s protocol for Gram-negative bacteria (Qiagen, Hilden, Germany). DNA concentrations were determined using the Qubit dsDNA BR assay kit (Invitrogen, Carlsbad, CA, United States). Genome sequencing was performed using the MiSeq instrument (Illumina, San Diego, CA, United States) at a 300 bp paired-end-read format. Raw reads were *de novo* assembled using the SPAdes v. 3.9.0 (Bankevich et al., 2012). The raw reads from the 12 *S*. Weltevreden strains were submitted to the European Nucleotide Archive under the project accession number PRJEB37452.

### Characterization and Phylogenetic and Comparative Genomics of *S.* Weltevreden

Assembled genomes of the 12 isolates were analyzed using the *Salmonella In Silico* Typing Resource (SISTR) (Yoshida et al., 2016) for confirmation of serovar. *In silico* MLST typing was performed with MLST 2.0 (Larsen et al., 2012). The sequences were further analyzed to identify acquired resistance genes by ResFinder v.3.2 (Zankari et al., 2012) and presence of known point mutations with 80% threshold for identity and 60% minimum alignment length. PlasmidFinder 2.1 (Carattoli et al., 2014) was used to identify plasmid replicons that are present in the genomes using default settings. Moreover, the assembled genomes were analyzed for identification of the main pathogenic markers on the *Salmonella* Pathogenicity Islands (SPIs) using SPI databases from CGE^1^ and the Pathogenicity Islands Database PAIDB v.2.0 (Yoon et al., 2015). The genomes were all analyzed for presence of prophages in PHASTER^2^ (Arndt et al., 2016), where the intact phage regions were reported as SWΦ.

For phylogenetic analysis, the raw reads of the 12 strains were analyzed using CSI-Phylogeny (Kaas et al., 2014) where single-nucleotide polymorphism (SNP) analysis was performed using the already described *S*. Weltevreden 10259 (Acc: SAMEA1904377) as reference (Makendi et al., 2016). To analyze the *S*. Weltevreden strains from China and Vietnam in a global context, the raw reads were further analyzed along with a collection of 60 *S.* Weltevreden strains. The global strains selected were all of the same sequence type ST-365 including 30 from the continental lineage and 30 from the island clonal line (Makendi et al., 2016) isolated from human, duck, pig, fish, poultry, seafood, environment, and vegetables (Supplementary Table S1). The obtained trees were annotated in iTOL (Letunic and Bork, 2016) and rooted with the stated reference strain.

To describe the genetic relationship between the environmental and clinical isolates, we performed a pangenomic analysis. In this analysis, 17 environmental strains (including our 12 strains and 5 additional strains isolated from seafood) and 17 clinical strains were used. The selected genomes for the pangenomic analysis were publicly available and part of the initial phylogenetic analysis and located on the continental clade, where our strains predominantly clustered (see “Results” and “Discussion” sections). All the 34 genomes analyzed were annotated using Prokka (Seemann, 2014) and the gff files were used as an input to the Roary (v.3.7.0) (Page et al., 2015) pangenome analysis tool in a Linux interface. The binary presence/absence data of accessory genes produced in Roary was used to calculate the associations between all genes in the accessory genome and the sample types of the isolates by employing the Scoary (v.1.6.11) tool (Brynildsrud et al., 2016). The accessory genome tree was visualized in phandango (Hadfield et al., 2018).

To decipher the potential genetic markers associated with the aquatic occurrence of *S.* Weltevreden, a number of known genes associated with aquatic bacteria were selected to make a database for local nucleotide BLAST using MyDbFinder 2.0. These include the salt resistance plasmids described in marine microorganisms (pSR1KM603475, pSR2KM603476, pSR4KM603478, pSR3KM 603477, pSR5KM603479, pSR6KM603480) (Mirete et al., 2015), as well as the SPI-2 encoded T3SS genes *sse*C and *ssa*U described to support survival of *S.* Typhi in *Acanthamoeba* in the aquatic environment (Bleasdale et al., 2009). Moreover, due to its previously reported role in the survival of *S.* Weltevreden in seafood, the chromosomally located *stn* gene (*Salmonella* enterotoxin) (Kumar et al., 2015; Ammar et al., 2016) was included in a locally built database. Using the SEED genome viewer from RAST (Overbeek et al., 2014), a functional gene comparison was made between clinical and environmental strains of *S.* Weltevreden. This included genes involved in carbohydrate biosynthesis, stress response as well as regulation and cell signaling established in the SEED subsystem annotation and previously reported to play a role in the survival of *Flavobacterium* spp. and *V*. *cholerae* in the aquatic environment (Kolton et al., 2013; Hounmanou et al., 2019a).

In addition, another genome-wide comparison was performed between our 12 *S*. Weltevreden strains and seven broad host range and host-adapted *Salmonella enterica* serovars including *S*. Typhi CT18, *S.* Typhimurium LT2, *S*. Enteritidis 92-0392, *S.* Dublin USMARC-69838, *S.* Pullorum QJ-2D-Sal, *S*. Gallinarum 287/91, and *S*. Choleraesuis SCSA50. The reference *S.* Weltevreden 10259 (SAMEA1904377), which is a clinical strain, served as reference to compare the genome content of *S.* Weltevreden with these other serovars using Blast Atlas in GView^3^. The functions of the obtained *S.* Weltevreden specific gene products were determined in the QuickGO Molecular function^4^ through UniProt (The UniProt Consortium., 2019).

## Results and Discussion

### Genetic Variations and Relatedness Among *S*. Weltevreden From Shrimp and Tilapia

**FIGURE 1:**
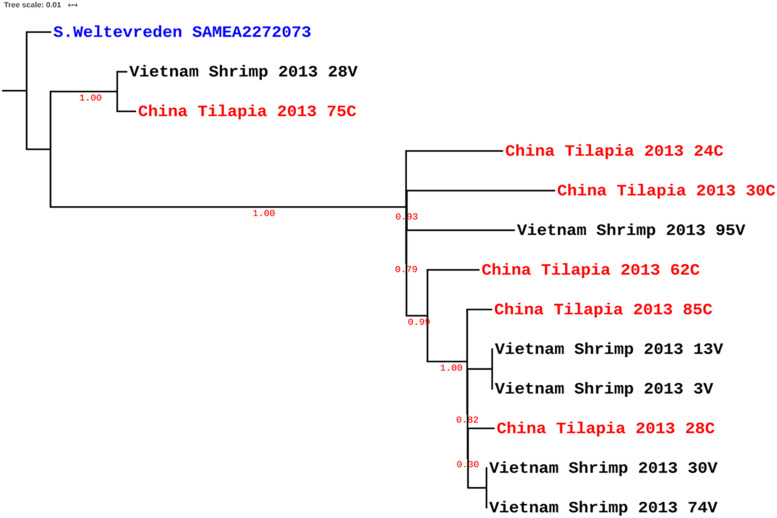
Maximum-likelihood SNP tree of *Salmonella* Weltevreden isolated from shrimp **(black)** in Vietnam and tilapia **(red)** in China. *S.* Weltevreden 10259 (SAMEA1904377; blue) served as reference to root the tree.

**FIGURE 2:**
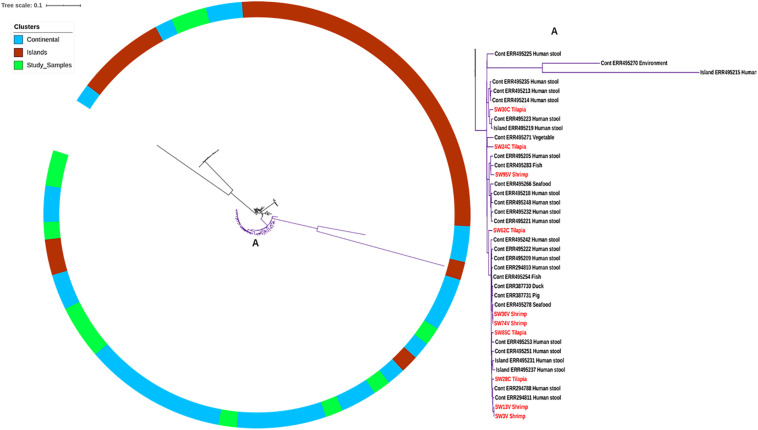
SNP tree showing *Salmonella* Weltevreden from this study (red font types in **A**) within a global context of the continental and island clusters (Makendi et al., 2016). **(A)** Depicts the continental clade where 10 of our 12 strains are clustered.

The strains characterized in this study were all of ST-365 and were previously isolated from tilapia aquaculture in China and shrimp culture sites in Vietnam without any known epidemiological association between strains and sites of isolation (Noor Uddin et al., 2015; Li et al., 2017). By PFGE fingerprinting, it was previously found that the strains isolated from the same source were closely related. We made a genomic comparison to get a better view of the relatedness of the isolates (Figure 1). This comparison showed that some strains isolated from shrimp in Vietnam are clonal with zero SNPs, e.g., 74V and 30V, as well as 3V and 13V (Figure 1). Other strains such as 24C, 30C, and 95V were located on the continental cluster and differed by up to 100 SNPs belonging to separate sub-clades as previously reported by Makendi et al. (2016) based on intrinsic genomic variations. As expected due to the higher discriminatory power of WGS compared with PFGE (Bayliss et al., 2017), other isolates with similar PFGE fingerprints were more diverse and had up to 312 SPNs within isolates from tilapia in China (Figure 1). Surprisingly, some strains isolated in shrimp from Vietnam were genetically related to strains recovered from tilapia in China, i.e., for instance 28V and 75C (20 SNPs), 30V and 28C (31 SNPs), and 13V and 85C (34 SNPs) (Supplementary Table S1). When analyzed with selected *S.* Weltevreden isolates from different parts of the world, the isolates predominantly clustered within a so-called continental lineage which constitutes mostly of isolates of clinical origin, with as low as seven SNPs difference, e.g., 30V versus Cont_ERR495254 (Figure 2 and Supplementary Table S1). The similarity among genomes of epidemiologically unrelated strains from shrimp and tilapia recovered in Vietnam and China suggests that the *S*. Weltevreden circulating in Southeast Asia may have a common source and recent spread, or they have a conserved genome. The observation corroborates their main clustering with other strains found predominantly in the Southeast Asian lineage of the continental cluster (Makendi et al., 2016). It should be noted that we did not select all available *S*. Weltevreden genomes, but a representative number of strains (selected across all countries, years of isolation, hosts, and sources of isolation) representing the continental and island clusters (Supplementary Table S1) from the previous study of Makendi et al. (2016) for the global localization of the genomes of our environmental strains from shrimp and tilapia.

### Genomic Characteristics of *S*. Weltevreden From Shrimp and Tilapia

The observed phylogenetic similarity between our environmental isolates and clinical strains from the continental Southeast Asian cluster is not surprising and indicates that our strains from shrimp and tilapia have the potential to cause disease in humans. This is further substantiated by the presence in our strains of main virulence-associated genes on the *Salmonella* Pathogenicity Islands (SPIs) 1–5, as well as a number of other pathogenicity islands (data not shown), which have been associated with virulence in humans and animals for *Salmonella* Typhimurium (Marcus et al., 2000). The presence of SPI1-5 known to be important for intestinal invasion indicates that the environmental isolates are capable of causing infection (Marcus et al., 2000; Lou et al., 2019) and invading the intestine. However, isolates of *S.* Weltevreden were previously reported to be significantly less invasive in Hep-2 cells but caused inflammatory reactions in mice (Makendi et al., 2016).

The *Inc*FII plasmid replicon is present in most of our strains (Table 1). This plasmid is part of a large family of *Inc*F plasmids that have been associated with *Salmonella* virulence and play a role during infection under low-iron conditions in host cells (Khajanchi et al., 2017). The closest reference to the *Inc*FII plasmid identified in our genomes is the highly transmissible plasmid pSPCV (CP000858) reported in the human pathogen *Salmonella* Paratyphi C encoding virulence genes such as the SPI2 effector genes of the *spv* gene clusters (Liu et al., 2009); however, the *spv* genes were not present in the genomes of *S.* Weltevreden.

**TABLE 1:** General properties and genetic characteristics of *S*. Weltewreden strains isolated from shrimp and tilapia.

Strain name^*a*^	Country of origin	Genome size (bp)	Number of contigs	n50	Resistance genes and mutations	Plasmid replicon
24C	China	4,969,946	140	197,156	aac(6′)-Iaa, parC p.T57S
28C	China	5,127,676	106	147,023	aac(6′)-Iaa, parC p.T57S	IncFII(S)
30C	China	4,865,581	94	175,961	aac(6′)-Iaa, parC p.T57S	–
62C	China	5,006,616	92	147,023	aac(6′)-Iaa, parC p.T57S	IncFII(S)
75C	China	4,983,794	146	111,941	aac(6′)-Iaa, *str*A, *str*B, *su*l2, *tet*(A), parC p.T57S	IncFII(S), IncQ1
85C	China	5,041,289	104	134,960	aac(6′)-Iaa, parC p.T57S	IncFII(S)
3V	Vietnam	5,075,193	132	134,960	aac(6′)-Iaa, parC p.T57S	IncFII(S)
13V	Vietnam	5,075,009	126	113,728	aac(6′)-Iaa, parC p.T57S	IncFII(S)
28V	Vietnam	4,969,248	76	143,942	aac(6′)-Iaa, parC p.T57S	IncFII(S)
30V	Vietnam	5,073,625	133	139,995	aac(6′)-Iaa, parC p.T57S	IncFII(S)
74V	Vietnam	5,087,031	120	108,166	aac(6′)-Iaa, parC p.T57S	IncFII(S)
95V	Vietnam	5,160,245	120	134,960	aac(6′)-Iaa, parC p.T57S	IncFII(S)

*^a^All strains from China were isolated from tilapia and the Vietnamese strains originated from shrimp.*

Altogether, our data indicate that the *S.* Weltevreden strains isolated in shrimp and tilapia are closely related to clinical strains with pathogenic potentials to cause infection in mammals. Moreover, our strains do not carry many antimicrobial resistance genes. Except for strain 75 C from tilapia, all the remaining strains only carried the aminoglycoside resistance gene *aac*(6′)-Iaa and presented a point mutation in *par*C p.T57S encoding resistance to quinolones, although they were originally reported susceptible to nalidixic acid and ciprofloxacin (Noor Uddin et al., 2015; Li et al., 2017). The discrepancy between susceptibility to gentamicin and the presence of the *aac(*6′*)-Iaa* gene is because the aac(6′)-Iaa enzyme does not acetylate gentamicin (Salipante and Hall, 2003). However, a previous study showed the presence of plasmid-located *aph*(3′)-Ia and strA/B genes conferring aminoglycoside resistance in *S*. Weltevreden from food products (Makendi et al., 2016); genes not found in our strains. Phenotypic resistance to quinolones seems increasingly to require both plasmid-mediated quinolone resistance genes like *qnr*A, *qnr*C, and two point mutations in the quinolone resistance-determining region such as parC and gyrA (Kotb et al., 2019). Point mutations in the *par*C gene alone are usually associated with a low increase in the minimum inhibitory concentrations to quinolones, so the discrepant results shown between our genomic and phenotypic observations are not surprising. Overall, the general susceptibility of *S*. Weltevreden to different antimicrobials suggests that it has a habitat and ecology with little if any exposure to antimicrobials, e.g., an aquatic habitat (Noor Uddin et al., 2015; Li et al., 2017).

The strain 75 C carried, in addition to the *par*C p.T57S mutation, *str*A, *str*B, *sul*2, and *tet*A resistance genes along with an *Inc*Q1 type plasmid on the same contig suggesting a plasmid-mediated resistance in this strain confirmed after blasting the contig. The identified *Inc*Q1 plasmid is similar to the pRSF1010 (M28829) of *E. coli* and the pNUC isolated in clinical *S.* Typhimurium conferring resistance to sulfamethoxazole, streptomycin, and tetracycline through the presence of *sul2*, *strAB*, and *tetA* genes (Oliva et al., 2017). Strain 75 C clusters with strains showing similar levels of antimicrobial resistance as shown for strains previously characterized and located in a continental sub-clade named “Vietnam Antimicrobial resistant” (Makendi et al., 2016). Nevertheless, in our study, this sub-clade of strains grouped on the island cluster, a discrepancy that we also found in a recent study which is probably caused by minor differences in the SNP calling methods (FastTree versus mpileup) (Minh et al., 2020).

Sequences of the phage VibrioΦ_X29 was found in strain 30C and the EnterobacterΦ_Tyrion phage in 95V, and the strains carried at least one of the common *Salmonella* prophages such as Gifsy_1 and 2, phiV10, sal3, Fels, PsP3, g341c, and SEN34 (Supplementary Table S2). The presence of the lysogenic phage VibrioΦ_X29 (Bhandare et al., 2017) in *S*. Weltevreden is interesting as it has not been reported previously in *S*. Weltevreden (Makendi et al., 2016) and deserves further attention. The finding suggests that *S.* Weltevreden has acquired the phage from *Vibrio* spp. which suggests that marine environments may be important ecological niches for this serovar.

It was also investigated if our environmental *S.* Weltevreden strains possess other genetic markers supporting their presence in marine environments including seafood such as salt tolerance. However, none of the strains carried genes and plasmids reported to encode salt resistance in marine microorganisms which may be due to the fact that the reference genes used for this analysis emanated from marine bacteria (Mirete et al., 2015), whereas *S.* Weltevreden may be less halophilic as it has also been isolated in freshwater aquaculture (Li et al., 2017). Thus, the absence of the high salt tolerance genes in our strains should be interpreted with caution because *S.* Weltevreden is often isolated in seafood.

Our strains possessed the SPI-2 encoded T3SS genes *sse*C and *ssa*U described to support survival of *S.* Typhi in *Acanthamoeba* in the aquatic environment (Bleasdale et al., 2009). Also, all our *S.* Weltevreden isolates harbor the chromosomally located gene *stn* which encodes the *Salmonella* enterotoxin Stn and has been shown to be upregulated during growth in seafood (Kumar et al., 2015); a gene which was found in all our strains. These genes are however not restricted to *S.* Weltevreden but are present in most *Salmonella enterica* serovars (Marcus et al., 2000; Ammar et al., 2016). The association of *S.* Weltevreden with aquatic environments is therefore difficult to document by comparative genomics alone because the strains causing disease in human are genetically similar to those in water which probably serves as their ecological niche. This observation is similar to the case of *V. cholerae* O1 found in aquatic environments where there was no genetic distinction between clinical and environmental strains isolated from Lake Victoria, although *V. cholerae* is known to have a tropism for the aquatic environment (Hounmanou et al., 2019a, b). Future laboratory experiments could assess the growth of *S.* Weltevreden in water and use proteomics to detect all differential expressed genes providing survival advantages to the bacterium. Similar to studies in *V. cholerae* and *S.* Typhi, the persistence of *S.* Weltevreden in the aquatic environment could also be assessed through various microbial interaction studies such as relationships with protozoa and other aquatic organisms like phytoplankton, zooplankton, and fish (Tezcan-Merdol et al., 2004; Douesnard-Malo and Daigle, 2011; Hounmanou et al., 2019c).

### The Accessory Genome of Isolated Strains in Comparison With Clinical Strains

**FIGURE 3:**
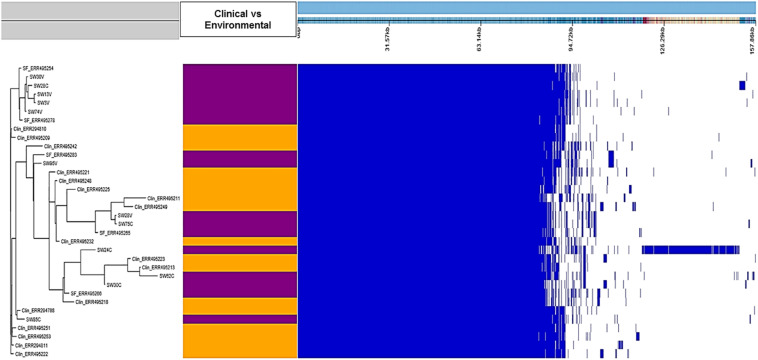
Presence–absence map of the genes in the pangenome of clinical and environmental *S.* Weltevreden. The tree to the left is the accessory binary tree where environmental strains are marked with purple whereas clinical strains are shown in orange color. The blue box marks the presence of a region whereas the white gaps represent the absent regions.

The genetic relatedness between our environmental and clinical *S*. Weltevreden strains revealed by the core genome–based phylogeny was also confirmed by the pangenome analysis. Roary alignment of the genomes in the clinical and environmental strains located in the continental clade where 10 of our 12 strains clustered revealed a pangenome size of 7891 genes including a core genome of 4892 genes which is similar to the number of core genes in most *Salmonella enterica* (Stevens et al., 2018). In a pangenome analysis, the accessory genome serves to reveal significantly unique genes for each clade. However, when scored and analyzed with Scoary commands, it was shown that the accessory genome content of the environmental strains was not significantly different from clinical strains with a Benjamini *p* > 0.05 between strains from the two origins (Supplementary Table S3 and Figure 3). Nevertheless, there was a unique genetic region in strain 24C compared with all the strains included in the analysis (Figure 3). This region was visualized using CLC genomic workbench and by the over 500 annotated proteins that are mostly additional copies of the already present genes in all the strains from the region (sheet 2 of Supplementary Table S3).

Following methodologies used to distinguish terrestrial and aquatic *Flavobacterium* spp. (Kolton et al., 2013), we performed a functional gene comparative genomic analysis with the SEED viewer in RAST (see “Materials and Methods” section). The findings of this analysis corroborated our pangenomic analysis demonstrating that all the SEED’s functional subsystems present in environmental strains were also found in clinical strains with similar proportions of gene content. For instance, functional genes involved in “virulence, disease, and defense”; “regulation and cell signaling”; “stress response”; and “carbohydrate biosynthesis” were evenly distributed in genomes from both sources. This further confirms the zoonotic potential of *S*. Weltevreden isolated in aquatic environments and seafood.

### Content Comparison of the Genome of *S*. Weltevreden to Seven Other *Salmonella enterica*

**FIGURE 4:**
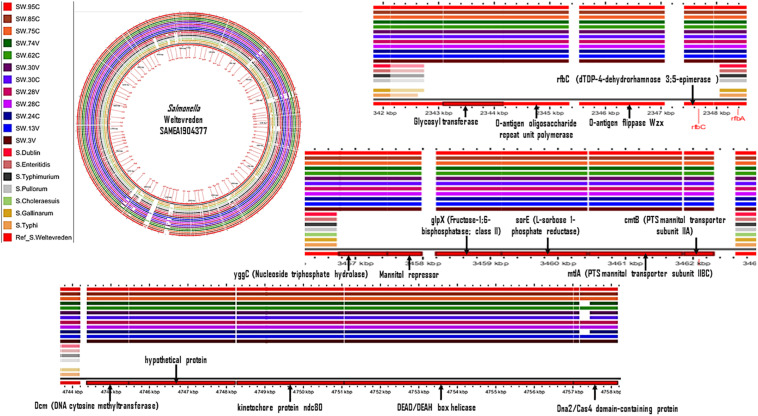
Genome content comparison of *S.* Weltevreden with *S*. Typhi, *S*. Typhimurium, *S*. Enteritidis, *S*. Dublin, *S*. Pullorum, *S*. Gallinarum, and *S*. Choleraesuis. Gaps in the genome show genetic regions potentially specific to *S.* Weltevreden.

In a further pangenomic analysis, we compared *S.* Weltevreden with genomes of seven other *Salmonella enterica* serovars, i.e., *S.* Typhi, *S.* Typhimurium, *S.* Enteritidis, *S.* Dublin, *S.* Pullorum, *S.* Gallinarum, and *S.* Choleraesuis. The objective was to detect genetic regions that are unique to *S.* Weltevreden and assess if the genes in such regions may play a role in aquatic niche adaptation. Various genetic elements were detected as specific to *S.* Weltevreden including *glp*X (Fructose-1;6-bisphosphatase; class II), *rfb*C (dTDP-4-dehydrorhamnose 3;5-epimerase), different PTS mannitol transporters, and sorE (L-sorbose 1-phosphate reductase) predominantly involved in the carbohydrate biosynthesis pathways (Figure 4 and Supplementary Table S4). Other unique elements were the O-antigen flippase Wzx which has an antiporter activity and xenobiotic transmembrane transporter activity present on the cell membrane (The UniProt Consortium., 2019). This analysis also detected DEAD/DEAH box helicases involved in various aspects of RNA metabolism, including nuclear transcription, pre-mRNA splicing, ribosome biogenesis, nucleocytoplasmic transport, translation, RNA decay, and organellar gene expression (The UniProt Consortium., 2019). Although these elements can potentially distinguish *S*. Weltevreden from other serovars, their role in aquatic niche adaption remains to be established. Furthermore, when blasted, the detected regions are also present in other bacteria such as *E. coli*, *Shigella* spp., *Mycobacterium leprae*, and *Pseudomonas* spp. (The UniProt Consortium., 2019). The identified *S*. Weltevreden specific elements are similar to the genetic markers found in the six genomic islands reported when *S*. Weltevreden was compared with other serovars including the DEAD-box ATP-dependent RNA helicase (*ydbR*), the mannose-specific phosphotransferase system (PTS), and the plasmid pSW82 (Acc. FR775255) (Brankatschk et al., 2012).

We are currently conducting growth experiments to verify findings in the genomic study. Preliminary data from these experiments show no differences between *S*. Weltevreden and *S*. Typhimurium LT2 when grown in media with different salt concentrations representing different aquatic environments from fresh to brackish water (unpublished data). However, increasing NaCl concentrations (0.5–6%) induced different growth responses within the *S*. Weltevreden populations. Moreover, because the genomic study revealed the presence of specific PTS systems in *S*. Weltevreden, we performed growth experiments with different concentrations of carbohydrates. The growth of *S.* Weltevreden in the minimal media M9 with glucose, mannitol, galactose, glycerol, and in combination glycerol + mannitol was compared with growth of *S.* Typhimurium TL2, *S.* Agona, and *S.* Seftenberg. The bioscreen showed that all tested strains were able to grow with the different tested carbon sources from 0.001 to 0.2% of carbohydrate with no difference between the growth rates (unpublished data). These preliminary results show no growth differences between *S.* Weltevreden and other *Salmonella* serotypes as opposed to the expectations from the genomic data and therefore points to the need for further growth experiments of other more specific carbon sources for PTS-specific system found in *S.* Weltevreden. This study revealed that the *S*. Weltevreden isolated from shrimp and tilapia in Vietnam and China possess pathogenic characteristics and are genetically closely related to clinical strains found in Southeast Asia. Antimicrobial resistance genes were uncommon. Comparative genomics indicate that the sources of human infections are likely fish, shrimp, and possibly other types of seafood. Various genomic regions were detected in the strains that differentiate them from other serovars and need to be further studied in experimental studies for their functional roles in the adaptation of *S*. Weltevreden to the aquatic environment. Moreover, the present study shows the need for new experiments describing aquatic reservoirs and microbial interactions that provide survival advantages to *S.* Weltevreden in aquatic environments. Genes that are overexpressed when *S.* Weltevreden is in water may also be determined. Growth experiments under different environmental conditions should also be considered.

## Data Availability Statement

The datasets presented in this study can be found in online repositories. The names of the repository/repositories and accession number(s) can be found in the article/Supplementary Material.

## Author Contributions

GU isolated the Vietnamese strains. AD, ML, TS, and GU wrote the first draft of the manuscript. YH performed the bioinformatics analyses with some guidance from PL and RH and revised and finalized the manuscript. AD, PL, RH, JO, and ML critically revised the manuscript to reach a final version. AD and ML conceived the study and provided funding. All authors revised and approved the final version of the manuscript.

## Conflict of Interest

The authors declare that the research was conducted in the absence of any commercial or financial relationships that could be construed as a potential conflict of interest.

## Footnotes

1 https://cge.cbs.dtu.dk/services/SPIFinder/

2 http://phaster.ca/

3 https://server.gview.ca/

4 https://www.ebi.ac.uk/QuickGO/

## Supplementary Material

TABLE S1 Public strains used and pairwise SNPs between genomes (Excel file).

TABLE S2 Prophages identified in the genomes of *S.* Weltevreden isolated in shrimp and tilapia from Vietnam and China, respectively.

TABLE S3 Content of the accessory genome in clinical and environmental strains (Excel file).

TABLE S4 Additional *S.* Weltevreden-specific genes when compared with *S*. Typhi, *S*. Typhimurium, *S*. Enteritidis, *S*. Dublin, *S.* Pollorum, *S*. Gallinarum, and *S.* Choleraesuis.
