# Supplementary material for: Molecular Characteristics and Zoonotic Potential of Salmonella Weltevreden From Cultured Shrimp and Tilapia in Vietnam and China
Source: Front Microbiol. 2020 Aug 25;11:1985. doi: 10.3389/fmicb.2020.01985 (PMC7477899; doi:10.3389/fmicb.2020.01985)
Supplement: TABLE S2 — Prophages identified in the genomes of S. Weltevreden isolated in shrimp and tilapia from Vietnam and China, respectively. [file Table_2.DOCX]

**Table S2.** Prophages identified in the genomes of *S.* Weltevreden isolated from shrimp and tilapia in Vietnam and China, respectively.

| **Strain** | **Phage Regions** | **Location in genome** | **Size** | **GC%** | **Intact PHASTER match** |
| --- | --- | --- | --- | --- | --- |
| 24c | SWΦ2 | Contig6: 97440-155584 | 58.1Kb | 50.73% | phiV10_NC_007804(34) |
|  | SWΦ3 | Contig8: 3-24606 | 24.6Kb | 52.13% | sal3_NC_031940(37) |
|  | SWΦ7 | Contig34: 2692-30820 | 28.1Kb | 51.28% | Gifsy_2_NC_010393(38) |
|  | SWΦ9 | Contig42: 84-19179 | 19Kb | 54.41% | Gifsy_1_NC_010392(18) |
| 28c | SWΦ2 | Contig10: 84843-127968 | 43.1Kb | 49.41% | sal3_NC_031940(28) |
|  | SWΦ4 | Contig15: 2880-40627 | 37.7Kb | 51.19% | Fels_1_NC_010391(15) |
|  | SWΦ5 | Contig16: 75290-114388 | 39Kb | 51.51% | sal3_NC_031940(21) |
|  | SWΦ6 | Contig25: 33511-68321 | 34.8Kb | 51.90% | PsP3_NC_005340(35) |
|  | SWΦ7 | Contig28: 18582-60308 | 41.7Kb | 47.33% | g341c_NC_013059(49) |
|  | SWΦ8 | Contig29: 717-40100 | 39.3Kb | 51.21% | SEN34_NC_028699(33) |
|  | SWΦ9 | Contig35: 2461-30589 | 28.1Kb | 51.27% | Gifsy_2_NC_010393(37) |
| 30c | SWΦ2 | Contig12: 3-45682 | 45.6Kb | 46.51% | Vibrio_X29_NC_024369(18) |
|  | SWΦ5 | Contig42: 3-17270 | 17.2Kb | 53.36% | Gifsy_2_NC_010393(20) |
| 62c | SWΦ2 | Contig5: 82083-167179 | 85Kb | 50.67% | phiV10_NC_007804(35) |
|  | SWΦ5 | Contig41: 3-22041 | 22Kb | 52.19% | Gifsy_2_NC_010393(27) |
|  | SWΦ7 | Contig45: 84-19179 | 19Kb | 54.41% | Gifsy_1_NC_010392(18) |
| 75c | SWΦ7 | Contig14: 76791-119725 | 42.9Kb | 51.90% | sal3_NC_031940(21) |
|  | SWΦ8 | Contig28: 33893-49313 | 15.4Kb | 55.91% | PsP3_NC_005340(22) |
|  | SWΦ10 | Contig44: 2692-24296 | 21.6Kb | 52.09% | Gifsy_2_NC_010393(26) |
| 85c | SWΦ2 | Contig5: 97440-155254 | 57.8Kb | 50.97% | phiV10_NC_007804(35) |
|  | SWΦ6 | Contig6: 33513-68323 | 34.8Kb | 51.90% | PsP3_NC_005340(35) |
|  | SWΦ7 | Contig29: 20237-54953 | 34.7Kb | 50.12% | sal3_NC_031940(28) |
|  | SWΦ9 | Contig43: 3-22041 | 22Kb | 52.19% | Gifsy_2_NC_010393(27) |
|  | SWΦ10 | Contig50: 3-12343 | 12.3Kb | 53.72% | Gifsy_1_NC_010392(11) |
| 3v | SWΦ6 | Contig26: 33513-68323 | 34.8Kb | 51.90% | PsP3_NC_005340(35) |
|  | SWΦ7 | Contig28: 18583-60369 | 41.7Kb | 47.37% | g341c_NC_013059(49) |
|  | SWΦ9 | Contig44: 269-20502 | 20.2Kb | 52.35% | Gifsy_2_NC_010393(24) |
|  | SWΦ11 | Contig52: 3-12343 | 12.3Kb | 53.72% | Gifsy_1_NC_010392(11) |
| 13v | SWΦ6 | Contig26:33513-68323 | 34.8Kb | 51.90% | PsP3_NC_005340(35) |
|  | SWΦ7 | Contig28: 18583-60369 | 41.7Kb | 47.37% | g341c_NC_013059(49) |
|  | SWΦ8 | Contig37: 1213-29341 | 28.1Kb | 51.27% | Gifsy_2_NC_010393(37) |
|  | SWΦ9 | Contig42: 84-24252 | 24.1Kb | 52.91% | SEN34_NC_028699(22) |
|  | SWΦ11 | Contig52: 84-12302 | 12.2Kb | 53.74% | Gifsy_1_NC_010392(11) |
| 28v | SWΦ5 | Contig12: 234-25242 | 25Kb | 49.71% | P4_NC_001609(5) |
|  | SWΦ7 | Contig17: 76790-119724 | 42.9Kb | 51.91% | sal3_NC_031940(21) |
|  | SWΦ9 | Contig27: 638-52720 | 52Kb | 51.45% | Gifsy_2_NC_010393(43) |
|  | SWΦ10 | Contig30: 132-15552 | 15.4Kb | 55.91% | PsP3_NC_005340(22) |
| 30v | SWΦ3 | Contig10: 20157-64508 | 44.3Kb | 49.60% | sal3_NC_031940(28) |
|  | SWΦ5 | Contig27: 33513-68323 | 34.8Kb | 51.91% | PsP3_NC_005340(35) |
|  | SWΦ6 | Contig29: 18583-60369 | 41.7Kb | 47.37% | g341c_NC_013059(49) |
|  | SWΦ7 | Contig38: 2692-30820 | 28.1Kb | 51.27% | Gifsy_2_NC_010393(38) |
|  | SWΦ8 | Contig41: 125-24293 | 24.1Kb | 52.91% | SEN34_NC_028699(22) |
|  | SWΦ9 | Contig51: 3-12343 | 12.3Kb | 53.72% | Gifsy_1_NC_010392(11) |
| 74v | SWΦ5 | Contig26: 33513-68323 | 34.8Kb | 51.91% | PsP3_NC_005340(35) |
|  | SWΦ6 | Contig28: 18583-60369 | 41.7Kb | 47.37% | g341c_NC_013059(49) |
|  | SWΦ7 | Contig40: 3-20245 | 20.2Kb | 53.17% | sal3_NC_031940(26) |
|  | SWΦ8 | Contig42: 3-22041 | 22Kb | 52.19% | Gifsy_2_NC_010393(27) |
|  | SWΦ10 | Contig53: 920-12320 | 11.4Kb | 54.36% | Gifsy_1_NC_010392(13) |
|  | SWΦ11 | Contig54: 3-11352 | 11.3Kb | 55.73% | Fels_1_NC_010391(11) |
| 95v | SWΦ1 | Contig1: 88034-148775 | 60.7Kb | 51.01% | sal3_NC_031940(39) |
|  | SWΦ4 | Contig9: 108031-148155 | 40.1Kb | 50.66% | Tyrion_NC_031077(32) |
|  | SWΦ6 | Contig26: 13608-48440 | 34.8Kb | 51.90% | PsP3_NC_005340(35) |
|  | SWΦ7 | Contig29: 20941-50235 | 29.2Kb | 51.37% | SEN34_NC_028699(23) |
|  | SWΦ9 | Contig43: 2692-23192 | 20.5Kb | 52.22% | Gifsy_2_NC_010393(25) |
|  | SWΦ10 | Contig49: 920-12320 | 11.4Kb | 54.35% | Gifsy_1_NC_010392(13) |
|  | SWΦ11 | Contig50: 63-11290 | 11.2Kb | 55.75% | Fels_1_NC_010391(11) |

**Table S4.** Additional *S.* Weltevreden-specific genes when compared to *S*. Typhi, *S*. Typhimurium, *S*. Enteritidis, *S*. Dublin, *S*. Pollorum, *S*. Gallinarum and *S*. Choleraesuis.

| **Genes** | **Gene products** | **Gene length (bp)** |
| --- | --- | --- |
| T4SS_Rhs | RHS repeat-associated core domain-containing protein | 596 |
|  | IS3 family transposase | 886 |
| IclR | IclR family transcriptional regulator | 686 |
|  | Molybdopterin-dependent oxidoreductase | 2333 |
| dmsB | 4Fe-4S ferredoxin | 518 |
| dmsC | dimethyl sulfoxide reductase subunit C | 788 |
|  | Tyrosine-type recombinase/integrase | 1187 |
| AlpA | phage transcriptional regulator | 197 |
| Rha | Rha family transcriptional regulator | 596 |
| Icd | host cell division inhibitor Icd-like protein | 554 |
| dnaG | DNA primase | 398 |
| ORF6N | ORF6N antirepressor | 794 |
| LysR | LysR family transcriptional regulator | 932 |
| YbhH | 4-oxalomesaconate tautomerase | 1055 |
|  | anion permease membrane protein | 1433 |
| acnA | Hydratase | 2258 |
| ptxA | Pertussis toxin; subunit 1 subfamily | 731 |
| MsgA | Virulence DinI family protein | 224 |
| pstS | Phosphate ABC transporter substrate-binding protein | 317 |
| pagC | Attachment invasion locus protein precursor | 557 |
| HxlR | Helix-turn-helix transcriptional regulator | 341 |
| kefG | NAD(P)H-dependent oxidoreductase | 557 |
|  | acyltransferase 3 | 1112 |
| fimD | Fimbrial outer membrane usher protein | 2480 |
| yehC | Fimbrial chaperone yehC | 680 |
|  | Fimbrial chaperone protein; partial | 545 |
|  | DUF2574 family membrane protein | 281 |
| ner | Transcriptional regulator | 182 |
| LexA | LexA family transcriptional regulator | 653 |
|  | HlyD family secretion protein | 1136 |
| lst | UDP-glucose--glucosyl LPS a 1; 2-glucosyltransferase | 941 |
|  | DeoR/GlpR transcriptional regulator | 758 |
| yfbT | HAD-IA family hydrolase | 647 |
